# Use of PARP inhibitors in prostate cancer: from specific to broader application

**DOI:** 10.3389/fendo.2023.1164067

**Published:** 2023-04-21

**Authors:** Zhenting Zhang, Lei Diao, Chao Zhang, Feifei Wang, Xin Guan, Xin Yao

**Affiliations:** ^1^ Department of Genitourinary Oncology, Tianjin Medical University Cancer Institute and Hospital, Tianjin, China; ^2^ PBG China Medical, Pfizer Inc, Shanghai, China

**Keywords:** prostate cancer, PARP inhibitor, DNA damage response and repair, novel hormonal therapy, metastatic prostate cancer

## Abstract

Prostate cancer (PC) is one of the major health issues of elderly men in the word. It is showed that there were approximately 1.414 million patients with PC in 2020 worldwide, with a high mortality rate in metastatic cases. In the present choices of treatment in PC, androgen deprivation therapy has long been as a backbone of them. But the clinical outcomes of patients with metastatic castration-resistant prostate cancer (mCRPC) were not ideal because of their poor prognosis, more effective therapeutic approaches are still necessary to further improve this problem. Poly (ADP-ribose) polymerase (PARP) inhibitors lead to the single-strand DNA breaks and/or double-strand DNA breaks, and result in synthetic lethality in cancer cells with impaired homologous recombination genes. It is estimated that approximately 20~25% of patients with mCRPC have a somatic or germinal DNA damage repair gene mutation. Furthermore, in “*BRCA*ness” cases, which has been used to describe as tumors that have not arisen from a germline *BRCA1* or *BRCA2* mutation, there were also a number of studies sought to extend these promising results of PARP inhibitors. It is worth noting that an interaction between androgen receptor signaling and synthetic lethality with PARP inhibitors has been proposed. In this review, we discussed the mechanism of action and clinical research of PARP inhibitors, which may benefit population from “specific” to the “all-comer” in patients with PC when combined with novel hormonal therapies.

## 1 Introduction

GLOBOCAN-2020 estimated that there were approximately 1.414 million total prostate cancer (PC) patients and 30.7/100,000 incidences worldwide (1). PC became the second most common malignancy in men, with a mortality of 6.8% (1). The prognosis of metastatic PC remains poor compared to the favorable clinical outcome of localized PC under today’s advanced healthcare system (2). Metastatic PC causes over 400 thousand deaths annually, and the number of deaths is expected to be more than doubled by 2040, according to the Global Burden of Disease study in 2016 (3).

PC can be categorized into hormone-sensitive prostate cancer (HSPC) and castration-resistant prostate cancer (CRPC) based on its response to hormonal therapy. Usually cancer progression to CRPC results in the death of the patients. In addition to androgen deprivation therapy (ADT), the existing management strategies for CRPC include the administration of PC vaccine, chemotherapy, anti-androgen therapy, radionuclide therapy, immunotherapy and targeted therapies such as poly (ADP-ribose) polymerase (PARP) inhibitors (2). Novel hormonal therapies (NHTs) have been tried in the treatment of metastatic HSPC (mHSPC) for many years, but no additional efficacious drugs against metastatic CRPC (mCRPC) are currently available, leaving the largest unmet treatment need for metastatic PC. Therefore, new drugs are eagerly sought for mCRPC to improve the clinical outcome of mCRPC.

PARP inhibitors are a new class of targeted drugs developed recently, which are used in the treatment of various tumors such as mCRPC, and they mainly inhibit tumor cells proliferation by damaging DNA. These drugs were initially approved for the treatment of breast or ovarian cancer and subsequently were used in the clinical management of PC. Globally, olaparib and rucaparib are presently available approved PARP inhibitors for the treatment of PC.

As clinical research advances, PARP inhibitors have not been limited to managing PC patients with *BRCA1/2* gene mutation and have been implemented in a wider range of patients with homologous recombination repair (HRR)-associated gene mutation. Notably, PARP inhibitors when combined with NHTs might further improve the efficacy of treatment against mCRPC. In this manuscript, the clinical progress of PARP inhibitors and their mechanism of action are described to explore their potential application in a wider type of mCRPC.

## 2 The action of PARP on cellular DNA

The two ways of cellular DNA damages are single-strand breaks (SSBs) and double-strand breaks (DSBs). DNA damage response and repair (DDR) mechanisms can rapidly detect DNA damage and repair cells from intrinsic and extrinsic injuries. The SSBs are restored by nucleotide excision repair, base excision repair, and base mismatch repair, whereas DSBs are repaired by HRR and non-homologous end joining (4). During the S-phase of the cell cycle, the DNA replication phase, homologous recombination (HR) uses sister chromatids as a reference to restore the nucleotides damaged in the replication fork arrest (5). Therefore, targeting the DDR process may provide valuable therapeutic options for certain conditions promoting carcinogenesis through DDR gene mutation. When tumor cells already have genomic defects, targeted tumor therapies probably address both the genetic defect and tumor effect, like a “double hit” (6).

PARP is a group of enzymes participate in the synthesis of poly (ADP-ribose) (PAR), which involved in several cellular processes. Among them, PARP-1 is the most widely expressed and abundantly found enzyme, and it is regarded as a key sensor protein for DNA damage. With its increased catalytic activity (500-fold) while responding to the damaged DNA (7), PARP-1 induces poly ADP-ribosylation (PARylation), i.e., cleaving nicotinamide adenine dinucleotide (NAD+) and moving the resulting ADP ribose to itself (autoPARylization) or other targeted proteins (PARylation). These post-translational modifications automatically activate PARP and other DNA repair enzymes, mediating DNA repair by modifying chromatin structure and by localizing DNA repair effectors (8, 9). PARP-1 and/or PARP-2 proteins can repair SSBs, and PARP-1 can also repair DSBs and replication damage (10).

*BRCA2* gene mutation is considered a high-risk factor for developing PC in men. About 20% of mCRPC patients displayed DDR-associated genetic variants, including *BRCA1/2* and *ATM* mutations. HRR is a *BRCA1/2* gene-dependent repair mechanism (11), and therefore, tumor cells carrying *BRCA1/2*-deficient genes cannot repair DNA damage through the HRR, and requiring PARP proteins for the restoration of SSBs (12). If PARP protein is inhibited by PARP inhibitors, DNA will not be repaired, and tumor cells will die subsequently.

## 3 Mechanism of action of PARP inhibitors

PC patients carrying *BRCA2* mutation have showed more effectively responded to carboplatin-based chemotherapy regimens than PC patients without *BRCA2* mutation (13). Carboplatin-based chemotherapy in the presence of DNA strand breaks caused by HRR-damage may produce synergistic lethal effects in tumor cells. These findings may help to understand the mechanisms of PARP inhibitors.

**Figure 1:**
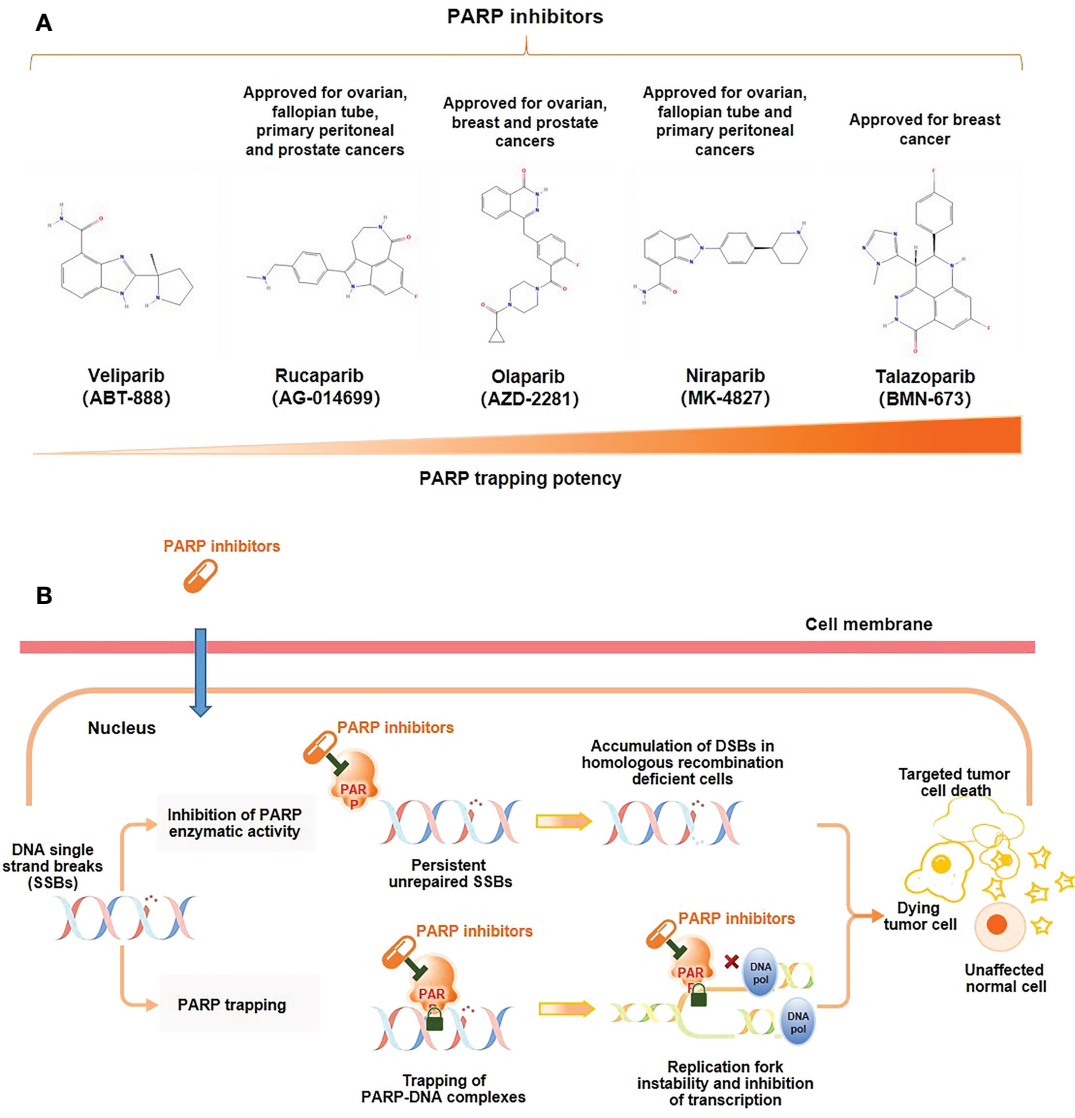
Mechanism of action of PARP inhibitors. **(A)** Molecular structure of PARP inhibitors and their capacity of trapping PARP; **(B)** PARP inhibitors lead to tumor cell death through two distinct mechanisms: the inhibition of the PARylation or trapping the PARP.

PARP inhibitors exert a pharmaceutically similar function to nicotinamide. They act primarily through two mechanisms (**Figure 1**) (1); inhibiting the catalytic activity of PARP by competitive binding to the active site against NAD+, hindering the repair of SSBs and thereby transforming into DSBs (10); (2) trapping PARP-1 into the damaged DNA through preventing PARP-1 release from DNA by inhibiting autoPARylization or enhancing DNA affinity to the catalytic site by creating allosteric PARP-1 structure (9). Further, PARP-1 delays the progression of replication forks, blocking the DSBs repair, and ultimately leading to cell death (12). PARP trapping does not occur independently of catalytic inhibition of PARylation. Since PARP-1 and PARP-2 cannot be isolated from DNA until PARP inhibitors dissociate from the active site following effective capture (10). Based on these mechanisms, *BRCA1/2* gene deficiency causing and PARP inhibition may synergistically prompt death in tumor cells, known as synthetic lethality (14).

*BRCA* mutated tumor cells are 1000-fold more sensitive to PARP inhibitors than *BRCA* wild-type cells (15). Hence, the development of PARP inhibitors initially emphasized the population with *BRCA1/2* gene mutation. However, with the advancement of molecular biological research, PARP inhibitor therapy has been gradually adopted for DDRs with mutations of *ATM, ATR, CHK1, CHK2, DSS1, RPA1, NBSI, FANCD2, FANCA, CDK12, PALB2, BRIP1, RAD51B, RAD51C, RAD51D, and RAD54*, in addition to *BRCA1/2* gene variants (16). PARP inhibitors also prolonged the survival in some cancer patients without HRR-associated genetic alterations as demonstrated in the PRIMA trial (17). In patients with advanced ovarian cancer responding to platinum-based chemotherapy, regardless of their HRR biomarker state, niraparib as a first-line maintenance therapy prolonged the progression-free survival (PFS), prompting the U.S. Food and Drug Administration (FDA) to approve the first PARP inhibitor therapy in April 2020 for the population without *BRCA* mutation (17). Nevertheless, the application potential of PARP inhibitors in tumor therapy needs to be comprehensively explored.

## 4 Pharmaceutical development and preclinical evaluation of PARP inhibitors for PC

PARP inhibitors that have been used clinically in the treatment of mCRPC are olaparib, rucaparib, niraparib, and talazoparib, and all of them can cause tumor cell death, but the mechanism of action varies between drugs.

No clinical trials have directly compared different PARP inhibitors, but preclinical studies have shown that PARP trapping capacity by different PARP inhibitors varies from strong to weak in a variety of tumor cells, including PC cells, talazoparib > niraparib > olaparib = rucaparib > veliparib (**Figure 1**) (18, 19). In human PC cells (DU145 cells), the levels of PARP-DNA complexes induced by talazoparib with concentration of 0.1 μM were comparable to those induced by olaparib with 10 μM (18). In human ovarian cancer cells (HeyA8 cells), talazoparib also showed the strongest activity of trapping PARP-1, and could be detected at subnanomolar concentrations, while olaparib showed significant capture of PARP-1 at its concentrations as low as 10 to 100 nM, which had a moderate trapping capacity (20). In addition, the capacity of trapping PARP-2 varies among PARP inhibitors, with more potent of niraparib and talazoparib than olapari (21, 22). Of these PARP inhibitors, current evidence suggests that talazoparib has the strongest trapping capacity for PARP, with about 100-fold higher than olaparib and rucaparib (18).

The ranking of trapping potency of PARP inhibitors described above is consistent with their cytotoxic potential. For instance, in most tumor cell lines, veliparib is still ineffective at concentrations up to 100 μM, while talazoparib is of potent cytotoxicity which showing effect even at nanomolar concentrations (18). The results from an indirect comparative analysis showed that the dissociation constant (Kd) to PARP of various PARP inhibitors and their monotherapy dose were not the same for similar efficacy (23). Among them, niraparib had the highest Kd for both PARP-1 and PARP-2, indicating its worst affinity for PARP. The dose of talazoparib as monotherapy was only 1 mg (od), while the doses of niraparib, olaparib, and rucaparib were hundreds of folds higher than that of talazoparib (300~600 mg, od/bd) (23). These may partially explain the close correlation between the clinical efficacy and safety of different PARP inhibitors and their trapping capacity.

The strong trapping potency of talazoparib for PARP may be related to its chemical structure. Among available PARP inhibitors at present, talazoparib has the most stable molecular structure with two racemic centers, reducing the occurrence of allosteric effects due to this unique stereostructure (10, 18, 24). The pharmacokinetic data of talazoparib showed a lower maximum concentration (C_max_), suggesting that it has a lower drug exposure compared to olaparib, niraparib and rucaparib *in vivo*. And talazoparib was found to have a relatively low half maximal inhibitory concentration (IC_50_) in PC cells. In addition, its relatively longer half-life is conducive to reducing the administration frequency (18, 24, 25) (**Table 1**). Together, talazoparib has shown certain advantages in preclinical studies in terms of both its trapping capacity and the inhibition against PARP enzymes.

**Table 1:** Pharmacodynamic/pharmacokinetic parameters of PARP inhibitors in solid tumors.

Drug	C_max_ (ng/mL)(25)	AUC (ng/mL·h)(25)	Mean T_1/2_ (h)(25)	PARP-1 Inhibition IC_50 (_nM)(24)	PARP Inhibition IC_50_* (nM) in PC(18)
**Olaparib**	5700~9500	33300~62100	6.52~11.9	1.94	18
**Niraparib**	1399~2071	19540~27852	36.2~36.45	/	/
**Rucaparib**	1940~2650	16900~47507	12.6	1.98	18
**Talazoparib**	16.4~32.84	208~244	50.0~50.73	0.57	11

* PARP inhibition in DU145 PC cell line.

## 5 Clinical development of PARP inhibitor monotherapies in PC

About 10% to 20% of PC patients develop mCRPC within 5 to 7 years of diagnosis (26). Metastatic PC is incurable, patients with metastatic PC have short survival, and the treatment goal is to prolong the survival of patients. Although today’s therapeutic advancement in hormonal therapy, chemotherapy and other healthcare provision are outstanding for mCRPC, the prognosis of patients remains unsatisfied and new therapeutic strategies are sought. PARP inhibitors have provided new treatment options for mCRPC in recent years.

PROfound phase III study based on two Phase II clinical trials, TOPARP-A (NCT01682772) and TOPARP-B (NCT01682772), elucidated mCRPC patients with HRR gene mutation and disease progression after prior treatment with enzalutamide or abiraterone acetate plus prednisone (AA). The results (data as of June 04, 2019) showed that, compared with the patients of the control group that treated with enzalutamide or AA, the patients treated with olaparib displayed longer radiographic progression-free survival (rPFS) (5.8 vs. 3.5 months, *P*<0.001) and better objective response rate (ORR) (22% vs. 4%; odds ratio 5.93, 95% CI: 2.01 -25.40). The incidence of grade ≥3 adverse events (AEs) was higher in the olaparib group than in the control group (27). Based on the outcomes of this study, the FDA approved the drug in May 2020 for the treatment of mCRPC harboring deleterious or suspected deleterious germline/somatic HRR gene mutation with disease progression after treatment with enzalutamide or AA in adult patients. The PROfound study is currently completed, but with detailed data analysis ongoing. Its final results would hopefully further confirm the benefits of olaparib in mCRPC.

GALAHAD (28) was a phase II clinical study with a single-arm design, which aimed to investigate the efficacy and safety of niraparib in patients with disease progression after prior paclitaxel and androgen receptor (AR)-targeted therapy. Its provisional results (as of May 23, 2019) showed that niraparib treatment gave rise to an ORR of 41%, a complete response rate (CRR) of 63%, a median rPFS and OS of 8.2 and 12.6 months in the *BRCA1/2* mutant population. At the same time, for the non-*BRCA* mutant cohort, a CRR of 17% was reported when treated by niraparib (28). The drug was then designated as a breakthrough therapy by the FDA in October 2019 for the treatment of mCRPC patients with a *BRCA1/2* mutation who have previously been treated with paclitaxel chemotherapy and AR-targeted therapy. The final results demonstrated niraparib’s significant anti-tumor activity in patients with DNA repair gene defect (DRD)-PC who had cancer progression after androgen signaling inhibitors and paclitaxel administration, particularly in patients with *BRCA* mutation, with controllable adverse events (29).

In the single-arm phase II TRITON-2 study (30) of rucaparib, patients with mCRPC, HRR-related gene mutations, and disease progression after 1-2 novel AR-targeted therapy and paclitaxel chemotherapy, the ORR was 43.5% and the prostate-specific antigen (PSA) response rate was 54.8% in the *BRCA1/2* mutation cohort. Anemia (25.2%) was the most common grade ≥3 adverse event. Rucaparib exhibited considerable anti-tumor activity and acceptable safety in treating patients with mCRPC harboring deleterious *BRCA* gene mutation (30). Consequently, the FDA accelerated the approval of rucaparib for the treatment of adult patients with mCRPC associated with deleterious *BRCA* mutation (germline and/or somatic) who have previously received AR-targeted therapy and paclitaxel chemotherapy in May 2020. The phase III randomized controlled trial (RCT) TRITON-3 study (NCT02975934), is currently in progress, substantiating the efficacy of rucaparib, and the outcome is yet to be published.

Although talazoparib has not been approved yet for PC, the phase II TALAPRO-1 study (31) evaluated its efficacy and safety. In mCRPC patients with DDR-HRR alterations and at least 1 prior dose of paclitaxel-based chemotherapy, talazoparib treatment achieved an ORR of 29.8% and a median follow-up of 16.4 months, with a significantly higher ORR in patients with *BRCA1/2* gene mutations (46%) than in those without *BRCA* gene mutations (25% for *PALB2* mutations, 12% for *ATM* mutations, and 0% for other mutations); the most common grades 3-4 AEs requiring emergency treatment were anemia (31%), thrombocytopenia (9%), and neutropenia (8%) (31). Suggestively, talazoparib showed durable anti-tumor activity in advanced mCRPC patients with DDR-HRR genetic alterations who have failed multi-line therapy, offering a new potential treatment option for patients with mCRPC (31).

PARP inhibitors treatment is generally considered safe, non-hematological toxicities such as fatigue, diarrhea, and nausea is common, but several grade 3-4 AEs may leading to limitations in treatment adherence. As shown in the previous trials, PARP inhibitors are associated with a significant increase in the risk of hematologic toxicities cancer patients (32). According to the results of a meta-analysis aimed at analyzing the adverse events in castration-resistant prostate cancer patients receiving PARP inhibitors, anemia was the most frequently observed grade 3-4 toxicity (24.1%), followed by thrombocytopenia (6.7%), neutropenia (5.2%), fatigue (4.9%), leukopenia (3.4%). And the incidence of treatment-related dose reduction and treatment discontinuation due to adverse events in mCRPC patients above was 26.9% and 14.1%, respectively (33). Therefore, frequent clinical monitoring still should be emphasized during PARP inhibitors administration.

Currently approved PARP inhibitors used as a monotherapy are only effective in PC patients with mutations in the *BRCA1/2* gene or HRR-related genes, but the incidence of these mutations is small with the rate of *BRCA1/2* mutations of 8.8% in mCRPC patients (34). Therefore, it is highly desirable to confirm the efficacy of PARP inhibitors in more PC populations without *BRCA1/2* mutation. In addition, patients with advanced PC may also become resistant to PARP inhibitors, as like other targeted therapies. Combination therapy with PARP inhibitors may be an important strategy for improving efficacy or resistance.

## 6 Mechanism of PARP inhibitors when combined with NHTs and their clinical development for PC

The results of clinical trials on olaparib, niraparib, rucaparib, and talazoparib have fully demonstrated that PARP inhibitors can benefit patients with mCRPC who carrying *BRCA1/2* gene or HRR-related gene mutation, and showed manageable safety (27, 29–31). The benefit populations from these medications include mCRPC patients with prior AR-based targeted therapy. Further, considering their mechanisms of action, AR inhibitors and PARP inhibitors may synergize in the treatment of mCRPC (35, 36). The efficacy of PARP inhibitors may also not limited to PC with *BRCA1/2* mutation (16, 17). Based on this, the combination of PARP inhibitors and inhibiting androgens by NHTs is considered to be a potential treatment strategy for the broader PC population, prompting extensive preclinical and clinical exploration by researchers.

### 6.1 Preclinical exploration of PARP inhibitors when combined with NHTs

AR signaling induces posttranslational modifications. AR contains >20 sites for modifications, such as phosphorylation, acetylation, SUMOylation, and ubiquitination, which involve in the development of PC (37). AR signaling regulates the expression levels of genes, such as *TMPRSS2* and *PSA* in normal prostate cells or PC cells (38). Therefore, repression of AR signaling is an important purpose of PC therapy. Androgen deprivation and anti-androgen therapy suppress the AR signaling and cause damage to the HR gene (e.g., *BRCA1/2* and *ATM*). Enzalutamide has been reported to induce a *BRCA*ness state (35). In addition to PARP1/2 enzymes, recent studies also found that ADP-ribosylation regulated AR signaling through PARP7-mediated nuclear pathways (37), and AR signaling also controlled PARP7 post-transcriptional regulation in PC cells (39). Based on this finding, a certain interaction between AR signaling and synthetic lethality caused by PARP inhibitors was proposed.

#### 6.1.1 Mechanisms of PARP affecting AR signaling

Polkinghorn et al. (40) found that approximately 32 direct target genes of AR, including PARP-1 and many DDR-related genes, were found in ChIP-sequencing analysis of AR in LNCaP cells, a human PC cell line, supporting the interaction of PARP inhibitors with AR signaling. *TMPRSS2*, an androgen-regulated gene and a product of repeated *ETS* gene fusion, is the driver of various cancers, including PC, with a *TMPRSS2* gene fusion to *ETS* transcription factors of 50% in PC^[37]^. PARP-1 interacted with *ERG* and inhibition of PARP-1 caused *ERG* overexpression-induced DSBs and inhibited the growth of ERG-positive PC cells (41). Schiewer et al. (42) also proposed that PARP-1 may contribute to PC progression through a dual role of DNA damage repair and transcription factor regulation, and clarified the following points (42) (1). PARP-1 can be recruited to the site of AR and can regulate the AR function; PARP-1 activity is increased in CRPC and can regulate the AR activity in castration-resistant states; (2) PARP inhibition can suppress the AR function, increase the sensitivity of PC cells to genotoxic damage, and synergize with anti-androgen therapy to inhibit cell proliferation, thereby suppressing tumor growth and delaying the development of the castration-resistant state. FOXA1 protein binds to chromatin and regulates AR transactivation by interacting with AR, Gui et al. (43)found that PARP-2 enhanced the AR activity through its interaction with FOXA1. In contrast, inhibition of PARP decreased AR gene expression and inhibited the AR-positive PC cellsgrowth (43). PARP promoted the recruitment of AR to the nucleus and the AR activity was decreased in PARP knockdown cells upon stimulation with the androgen dihydrotestosterone and restored following PARP-1 supplementation (42). Gui et al. (43) also found that targeted PARP blockade impaired the AR function through interaction with the transcriptional activator FOXA1. Hence, PARP has the potential to enhance AR activity and inhibition of PARP decreases AR activity, provoking NHTs to benefit further.

#### 6.1.2 Mechanism of NHTs affecting PARP activity

Genes involved in DNA damage repair may be downregulated following AR knockdown (36), demonstrating that NHTs has the potential to induce a phenotype resembling HRR deficiency and enhance the inhibiting efficacy of PARP-1. PARP-1 activity was significantly increased in PC patients after initiation of ADT compared with pre-treatment (36), also suggesting that inhibition of AR is associated with the up-regulation of PARP. PARylation, representing the PARP-1 activity, was elevated in the ADT-resistant PC cells compared with ADT-sensitive PC cells (42). Codeletion of *BRCA2* and *RB1* was found in approximately 10% to 50% of PC patients, and the codeletion might be one of the resistance mechanisms in NHTs (44); the sensitivity to PARP inhibition was increased in this state, and the inhibition of PARP might have weakened the growth of mCRPC cells (44). Therefore, early intervention using combined PARP inhibition and NHTs may delay the prospective resistance in patients with metastatic PC.

#### 6.1.3 Translational studies of PARP inhibitors when combined with anti-neoplastic drugs

Li et al. (35) showed that enzalutamide, olaparib, and their combination downregulated the HR-related genes (*BRCA1, RAD51AP1, RAD51C, RAD54L*, and *RMI2*) in AR-positive PC cells, using the strategy of synergizing enzalutamide and olaparib activities in increasing PC cell apoptosis and inhibiting cell growth (35). Further, olaparib plus enzalutamide treatment inhibited PC tumor growth in subcutaneous patient-derived xenograft tumor models (MDA PCa 133-4, AR-positive) as well as in two orthotopic PC cell lines (AR-positive VCaP and CWR22Rv1)^[34]^. In a study by Asim et al. (36), the cell viability was significantly reduced in AR-positive PC cell lines, C4-2 and LN3, after olaparib plus bicalutamide/enzalutamide treatment. Combined inhibition of AR and PARP by olaparib plus enzalutamide decreased the proliferation of PC cells in xenograft PC model mice after 1 week of treatment, suggesting that the combination may synergistically inhibit tumor growth (36).

### 6.2 Clinical research progress of PARP inhibitors when combined with NHTs

In 2018, the phase II NCI 9012 study (45) showed negative results with the PARP inhibitor veliparib plus AA regimen initially, but on further analysis, this combination regimen resulted in a significantly higher PSA response (90% vs. 56.7%, *P*=0.007) and longer PFS (14.5 vs. 8.1 months, *P*=0.025) compared with the control group without veliparib in patients with DRD-mutated mCRPC. Although this is a feasible combination regimen, the complexity and biological context of mCRPC patients should be considered in future clinical trial designs (45). Another phase II RCT study evaluated the olaparib plus AA in patients with mCRPC and found that this regimen prolonged the median rPFS compared with the placebo plus AA group (13.8 months vs. 8.2 months, *P*=0.034), proposing that this regimen may provide additional clinical benefit to a wider range of patients with mCRPC (46). These patients were not selected on the basis of biomarker criteria, suggested that they might benefit from the combination treatment irrespective of HRR mutation status (46). The PROpel study (47) presented at the 2022 annual meeting of the American Society of Clinical Oncology (ASCO) further demonstrated that olaparib plus AA, as the first-line therapy for mCRPC (including patients with HRR mutated and HRR-non-mutated), significantly prolonged rPFS in patients compared with the placebo plus AA group (24.8 vs. 16.6 months, *P*<0.0001). Preliminary results from the MAGNITUDE study (48) reported that niraparib plus AA significantly improved rPFS (relative risk 0.53, 95% CI: 0.36-0.79, *P*=0.0014) and reduced the risk of disease progression/death (47% vs. 27%) in the *BRCA1/2* mutation subgroup of mCRPC.

The success of the olaparib/niraparib plus AA regimen may have been attributed to the more potent PARP trapping ability of olaparib/niraparib compared with veliparib plus AA (16), suggesting that it is critical to select suitable PARP inhibitors when designing combination therapies. Talazoparib is a PARP inhibitor exhibiting a dual mechanism of action that may synergize with NHTs activity (24). On the one hand, talazoparib inhibited PARP catalytic activity and displayed potent PARP trapping ability. On the other hand, talazoparib monotherapy demonstrated potent anti-tumor activity in HRR-deficient mCRPC patients. Recent studies demonstrated that enzalutamide not only inhibited the androgen binding to AR but also suppressed the AR nuclear translocation, as well as AR-mediated DNA binding in anti-androgen treatments (49–51). Enzalutamide showed improved efficacy compared with abiraterone as the first-line treatment in patients with mCRPC (52, 53). Therefore, enzalutamide is a preferred drug for NHTs when combined with a PARP inhibitor. As of January 2023, several ongoing clinical trials using this combination strategy were identified in the *ClinicalTrials.gov* database, and their results are anticipated (**Table 2**). It has been found that HRR gene alterations are associated with the worse outcomes in mHSPC patients, with significantly shorter time to mCRPC (54). The results supported the possibility that using PARP inhibitors earlier in the clinical course for PC patients. And as shown in **Table 2**, the regimen of PARP inhibitors in combination with NHTs may bring promising results for HSPC or PC patients.

**Table 2:** Ongoing clinical trials with the combination of PARP inhibitors and NHTs in PC.

Drug	Subjects	Study Registration No./Title	Study Design	Study Phase	Treatment & Grouping	Primary Outcomes	Study Progress
**Olaparib**	mHSPC (carry deleterious germline or HRR mutations)	NCT05167175/ PROact	Single Group Assignment	Phase II	Olaparib+AA	rPFS	Recruiting
	mCRPC	NCT01972217/ D081DC00008	RCT	Phase II	Olaparib+AA vs Placebo+AA	rPFS; Percentage of Patients with Progression Events or Death	Active, not recruiting
	mCRPC	NCT03012321/ NU_16U05	RCT	Phase II	AA vs. Olaparib vs. Olaparib+AA	Objective PFS	Recruiting
	mCRPC	NCT05171816/ D081SC00001Sub	RCT	Phase III	Olaparib + AA vs. Placebo+ AA	rPFS	Active, not recruiting
	mCRPC	NCT03732820/ PROpel	RCT	Phase III	Olaparib+AA vs. Placebo+AA	rPFS	Active, not recruiting
**Niraparib**	PC	NCT04194554/ ASCLEPIuS	Single Group Assignment	Phase I, II	AA+Leuprolide+100 mg/200mg Niraparib but held for 5 days (+/- 2 days) prior to RT, during SBRT, and 5 days (+/- 2 days) after last fraction of SBRT AA+Leuprolide+200 mg Niraparib without breaks during SBRT until completion of 6 cycles	DLT; Proportion of patients experiencing biochemical failure	Recruiting
	deleterious germline or somatic HRR gene-mutated mHSPC	NCT04497844/ AMPLITUDE	RCT	Phase III	Niraparib+AA vs. Placebo+ AA	rPFS	Recruiting
	mCRPC	NCT04577833/ CR108783	RCT	Phase I	Treatment: Niraparib Formulation 1: A; Niraparib Formulation 2: B; Niraparib Formulation 3: C; Niraparib Formulation 4: D; Cohort: AA+Treatment ABD; AA+Treatment ADB; AA+Treatment CBD; AA+Treatment CDB	C_max,ss_; AUC_(0-24h),ss_; Ratio of Individual C_max,ss_ Values; Ratio of individual AUC _(0-24h),ss_ Values	Active, not recruiting
	mCRPC	NCT03748641/ MAGNITUDE	RCT	Phase III	Treatment: Phase RCT: Niraparib+AA vs. Placebo+ AA; Phase OLE:all receive Niraparib+AA Cohort 1: Participants with mCRPC and HRR Gene Alteration; Cohort 2: Participants with mCRPC and No HRR Gene Alteration; Cohort 3 (Open-label): Participants with mCRPC	rPFS	Active, not recruiting
**Rucaparib**	CRPC and mPA and phase IV/IVA/IVB PC	NCT04455750/ CASPAR	RCT	Phase III	Rucaparib+Enzalutamide vs. Placebo+Enzalutamide	rPFS;OS	Recruiting
	mCRPC	NCT04179396/ RAMP	Non-Randomized, Parallel Assignment	Phase Ib	Rucaparib+Enzalutamide vs. Rucaparib+AA	PK; treatment-related AEs/SAEs	Active, not recruiting
**Talazoparib**	HSPC	NCT04734730/ 20476	Single Group Assignment	Phase II	Talazoparib+AA+ADT	PSA nadir<0.2	Recruiting
	mHSPC	NCT04332744/ ZZ-First	RCT	Phase II	Talazoparib+Enzalutamide+ADT vs. Enzalutamide+ADT	PSA-CR	Recruiting
	DDR-deficient mHSPC	NCT04821622/ TALAPRO-3	RCT	Phase III	Talazoparib + Enzalutamide vs. Placebo + Enzalutamide	rPFS	Recruiting
	mCRPC	NCT03395197/ TALAPRO-2	RCT	Phase III	Talazoparib+Enzalutamide vs. Placebo+Ezalutamide	Confirm the dose of Talazoparib (part 1); rPFS(part 2)	Active, not recruiting

RCT: Randomized controlled trial; OLE, Open label extension; PC, Prostate cancer; mCRPC, Metastatic castration resistant prostate cancer; mHSPC, Metastatic hormone-sensitive prostate cancer; AA, Abiraterone Acetate plus Prednisone; rPFS, Radiographic progression-free survival; OS, Overall survival; ADT, Androgen deprivation therapy; SBRT, Stereotactic body radiotherapy; DLT, Dose Limiting toxicities; MTD, Maximum Tolerated dose; RP2D, Recommended Phase 2 Dose; PPSA, Prostate specific antigen; CR, Complete response.

## 7 Mechanisms of PARP inhibitor actions when combined with other personalized therapies and their clinical development for PC

### 7.1 PARP inhibitors when combined with immunotherapies

In preclinical studies, PARP inhibitors upregulated the programmed cell death 1 ligand 1 (PD-L1) expression and enhanced the tumor-associated immunosuppression, but PARP inhibitors when combined with anti-PD-L1 therapy were more effective (55). In a non-randomized phase I/II clinical trial, olaparib when combined with the immune checkpoint inhibitor durvalumab achieved an rPFS of 16.1 months in patients with mCRPC, with an rPFS rate of 51.5% within 1 year. Further, most patients with *BRCA2* mutation showed a good response (rPFS rate of 83.3% within 1 year) (56). Based on the interim results of another phase Ib/II KEYNOTE-365 study, the combination therapeutic regimen improved response in patients with mCRPC (regardless of HRR state) compared with olaparib or pembrolizumab monotherapy, with ORRs of 33% and PSA response rates of 50% in *BRCA-*mutated patients (57). And in the *BRCA*-non-mutated cohort, patients have likewise obtained a certain response, with ORRs of 6% and PSA response of 14% (57).

### 7.2 PARP inhibitors when combined with other targeted therapies

PARP inhibitors were combined with therapies against vascular endothelial growth factor (VEGF). PARP inhibitors exerted some anti-angiogenic effects in invasive PC cells *in vitro*, downregulated VEGF expression, and induced PC cell apoptosis (58). A phase II RCT further confirmed a significant increase in median rPFS in patients with mCRPC who received olaparib plus cediranib compared with olaparib monotherapy (8.5 vs. 4.0 months, *P*=0.033) (59). In addition, combinations of PARP inhibitors and PI3K/AKT inhibitors also improved the treatment efficacy, in which PI3K signaling promoted the DNA double-strand repair through interaction with HRR complexes and inhibition of PI3K enhanced the anti-tumor effect of PARP inhibitors (60). An ongoing Phase Ib clinical trial is evaluating rucaparib plus AKT inhibitor ipatasertib (NCT03840200), and its results will provide a reference regarding this regimen.

### 7.3 Other combinatory regimens at the clinical trial phase

In addition to drug therapies, clinical studies investigated PARP inhibitors combined with other treatment modalities. The NADIR study (NCT04037254) combined the radiotherapy and the LuPARP study (NCT03874884) and the COMRADE study (NCT03317392) incorporated the radionuclide therapy. The outcomes of these studies will support wider application strategies of PARP inhibitors.

## 8 Looking into the future

In recent years, evidence-based elucidations demonstrated the efficacy and safety of PARP inhibitors as monotherapy in mCRPC with *BRCA 1/2* gene mutation or HRR-related gene mutation. A comprehensive investigation of PARP inhibitors has not been paused, and over 80 PARP inhibitor treatment studies in PC patients, including a variety of monotherapies or combination regimens, are found in the database of *ClinicalTrials.gov*. The research and development of combination therapies will enhance treatment efficacy, improve drug resistance, and address other issues. Notably, PARP inhibitors combined with NHTs are expected to provide benefits to a wider population with PC.

The following directions specific to PARP inhibitors are worthy of further exploration: (1) exploring potential response biomarkers to PARP inhibitor treatment to determine the precise indicative population; (2) selecting different PARP inhibitors in combination therapies may have different resistance mechanisms and further exploration to overcome resistance is needed; (3) novel hormonal therapy is very effective in both mHSPC and mCRPC, and adding PARP inhibitors may further improve the cancer prognosis; (4) translating preclinical results into clinical applications should be focused. When these issues are addressed, adding PARP inhibitors to the treatment protocols could provide better survival in patients with PC and other cancers in the future.

## Author contributions

ZZ and XY contributed to the conception, design, and final approval of the submitted version. LD and CZ contributed to completing the Figure, writing the paper. All authors contributed to the article and approved the submitted version.
